# Activation of CTNNB1 by deubiquitinase UCHL3-mediated stabilization facilitates bladder cancer progression

**DOI:** 10.1186/s12967-023-04311-3

**Published:** 2023-09-22

**Authors:** Tao Liu, Meng-Qi Fan, Xiao-Xiao Xie, Qi-Peng Shu, Xue-Hua Du, Lin-Zhi Qi, Xiao-Dong Zhang, Ming-Hui Zhang, Guang Shan, Run-Lei Du, Shang-Ze Li

**Affiliations:** 1 School of Medicine, Chongqing University, Chongqing, 400030 China; 2 Department of Urology, Zhongnan Hospital of Wuhan University, Wuhan, 430071 Hubei China; 3 Hubei Key Laboratory of Cell Homeostasis, College of Life Sciences, Wuhan University, Wuhan, 430072 Hubei China; 4 Department of Urology, Renmin Hospital of Wuhan University, Wuhan, 430060 Hubei China; 5 Department of Obstetrics and Gynecology, The First Affiliated Hospital of Zhengzhou University, Zhengzhou, 450052 Henan China

**Keywords:** UCHL3, Bladder cancer, Wnt signaling pathway, CTNNB1

## Abstract

**Background:**

The catenin beta 1 gene (CTNNB1) plays a crucial role in the malignant progression of various cancers. Recent studies have suggested that CTNNB1 hyperactivation is closely related to the occurrence and development of bladder cancer (BCa). As a member of the deubiquitinating enzyme (DUB) family, ubiquitin C-terminal hydrolase L3 (UCHL3) is abnormally expressed in various cancers. In this study, we discovered that UCHL3 is a novel oncogene in bladder cancer, suggesting it is a promising target against bladder cancer.

**Methods:**

We utilized CRISPR‒Cas9 technology to construct cell lines with UCHL3 stably overexpressed or knocked out. The successful overexpression or knockout of UCHL3 was determined using Western blotting. Then, we performed CCK-8, colony formation, soft agar and Transwell migration assays to determine the impact of the UCHL3 gene on cell phenotype. RNA-seq was performed with UCHL3-depleted T24 cells (established via CRISPR–Cas9-mediated genomic editing). We analyzed differences in WNT pathway gene expression in wild-type and UCHL3-deficient T24 cell lines using a heatmap and by gene set enrichment analysis (GSEA). Then, we validated the effect of UCHL3 on the Wnt pathway using a dual fluorescence reporter. We then analyzed the underlying mechanisms involved using Western blots, co-IP, and immunofluorescence results. We also conducted nude mouse tumor formation experiments. Moreover, conditional UCHL3-knockout mice and bladder cancer model mice were established for research.

**Results:**

We found that the overexpression of UCHL3 boosted bladder cancer cell proliferation, invasion and migration, while the depletion of UCHL3 in bladder cancer cells delayed tumor tumorigenesis in vitro and in vivo. UCHL3 was highly associated with the Wnt signaling pathway and triggered the activation of the Wnt signaling pathway, which showed that its functions depend on its deubiquitination activity. Notably, Uchl3-deficient mice were less susceptible to bladder tumorigenesis. Additionally, UCHL3 was highly expressed in bladder cancer cells and associated with indicators of advanced clinicopathology.

**Conclusion:**

In summary, we found that UCHL3 is amplified in bladder cancer and functions as a tumor promoter that enhances proliferation and migration of tumor cells in vitro and bladder tumorigenesis and progression in vivo. Furthermore, we revealed that UCHL3 stabilizes CTNNB1 expression, resulting in the activation of the oncogenic Wnt signaling pathway. Therefore, our findings strongly suggest that UCHL3 is a promising therapeutic target for bladder cancer.

## Introduction

Bladder cancer (BCa) is a highly life-threatening urogenital neoplasm, posing a significant societal burden with a reported 81,400 new cases and 17,980 BCa-related deaths in the USA in 2020 [1] [2]. Environmental factors like cigarette smoking and occupational exposure to paint components contribute to the development of bladder cancer [3, 4]. Current standard interventions, including surgical resection, immunotherapy, and chemotherapy, are effective for early-stage patients [5]. However, the majority of patients are diagnosed at an advanced stage. Genomic alterations have been increasingly recognized as key factors in bladder cancer [6]. Thus, investigating the molecular mechanisms underlying bladder cancer pathogenesis and identifying new drug targets are essential.

The Wnt signal transduction pathway is a canonical signaling pathway that is reported to be frequently activated abnormally in human cancers [7]. In the context of bladder cancer, growing evidence suggests that aberrant Wnt pathway activation plays a crucial role in its pathology [8].A key factor in its activation is the downstream transcription coactivator CTNNB1, and its sustained accumulation in the cytoplasm is critical to the activation of the Wnt pathway [9]. In most malignancies, a decline in cytoplasmic catenin levels triggers the obstruction of Wnt/β-catenin signaling, inhibiting cancer progression [10, 11]. The phosphorylation- and ubiquitylation-dependent proteasomal degradation of CTNNB1 is a reasonable mechanism to control the activity of CTNNB1 in cells and the cancerogenesis of cells [12–15]. For instance, TRAF6 (TNF receptor associated factor 6) promotes the interaction between LC3B and CTNNB1 through the ubiquitination of LC3B and promotes the selective autophagic degradation of CTNNB1, inhibiting colorectal cancer metastasis [16]. COP1, an E3 ubiquitin ligase, can modulate CTNNB1 levels by affecting its ubiquitination-mediated degradation [17]. While the ubiquitination process of CTNNB1 has been well studied, there is a scarcity of research on CTNNB1 deubiquitinating enzymes.

Studies have demonstrated that deubiquitinases are potential mediators of cancerogenesis and neurodegeneration [18–20]. UCHL3, a member of the deubiquitinase enzyme family, belongs to a group of proteases that catalyze the removal of ubiquitin from polypeptides [21]. The UCHL3 gene is located at 13q22.2 and carries 13 exons. This protein may hydrolyze the ubiquitinyl-N-epsilon amide bond of ubiquitinated proteins to release ubiquitin for use in another catalytic cycle. This deubiquitinase (DUB) regulates posttranslational modification and thus regulates cellular homeostasis [18]. For example, UCHL3 contributes to non-small cell lung cancer cell growth by stabilizing TRAF2 (TNF receptor-associated factor 2) [22]. UCHL3 maintains aryl hydrocarbon receptor (AhR) protein stability and confers cancer stem-like properties to non-small cell lung cancer cells, functioning as a tumor promoter [21]. However, whether UCHL3 is involved in the deubiquitination of CTNNB1 and its role in bladder cancer remain unknown.

The aim of this study is to elucidate the mechanisms underlying the impact of UCHL3 on the initiation and progression of bladder cancer and to identify potential targets for its treatment.

## Methods

### Reagents and antibodies

Mouse monoclonal antibodies against FLAG (Cat. #M085-3, MBL), HA (Cat. #M0291-3, MBL), and GAPDH (Cat. #60004-1-Ig, Proteintech); rabbit monoclonal antibodies against CTNNB1 (Cat. #8480, CST), GFP (Cat. #50430-2-AP, Proteintech), UCHL3 (Cat. #ab241490, Abcam), and C-MYC (Cat. #ab32072, Abcam); rabbit polyclonal antibodies against AXIN2 (Cat. #20540-1-AP, Proteintech), Ki67 (Cat. #27309-1-AP, Proteintech), LEF1 (Cat. #50430-2-AP, Proteintech), Cyclin D1 (Cat. #50430-2-AP, Proteintech), ACTB (Cat. #50430-2-AP, Proteintech); and antibodies against the protein translation inhibitor cycloheximide (CHX, Cat# C7698, Sigma) and the proteasome inhibitor MG132 (Cat. #S2619, Selleckchem) were used in this study.

### Cell culture

The EJ and T24 human bladder cancer cell lines were cultured in McCoy's 5A medium (AppliChem, Darmstadt, Germany) containing 10% fetal bovine serum (FBS; HyClone, Logan, UT, USA) and 100 U of penicillin–streptomycin (Gibco, Carlsbad, CA, USA). HEK293 T cells were cultured in Dulbecco’s modified Eagle’s medium (DMEM) (Monad) supplemented with 10% FBS and 100 U of penicillin‒streptomycin. The EJ, T24 and HEK293 T cells were purchased from the Cell Storage Center of Wuhan University (Wuhan, China) and were incubated in a 5% CO_2_ incubator at 37 °C.

### Transient transfection and lentivirus-mediated stable overexpression

To overexpress UCHL3, UCHL3 cDNA from 293 T cells was amplified by PCR and then ligated into a pHAGE-puro-3 × Flag plasmid. To abrogate the deubiquitinating activity of UCHL3, we replaced the active Cys94 residue in UCHL3 with an Ala residue, and the resulting cDNA UCHL3^C94A^ construct was inserted into a pHAGE-puro-3 × Flag plasmid. The recombinant vectors carrying UCHL3^C94A^ or UCHL3 were transfected into 293 T cells with the packaging plasmids pVSVg (AddGene 8454) and psPAX2 (AddGene 12260). Forty-eight hours later, the medium containing lentiviral particles was harvested, filtered and subjected to ultracentrifugation. EJ cells at 85% confluence were infected with prepared viruses at the multiplicity of infection (MOI) of 0.2. Forty-eight hours post-infection, EJ cells were treated with puromycin (1 μg/mL) for 7 days, and the surviving cells were pooled for use in Western blotting.

### Genetic knockout of UCHL3 in T24 cells

To abrogate UCHL3 in T24 cells, we used the CRISPR‒Cas9 gene-editing technique, and sgRNA sequences targeting *UCHL3* Exons 1 and 2 (sgRNA1 and sgRNA2) were synthesized by Tianyi Huiyuan Biotechnology Company (Wuhan, China). The prepared sgRNA oligos were ligated into a lentiCRISPRv2 plasmid (Addgene, USA). The recombinant lentiCRISPRv2-sgRNAs were packaged and amplified in 293 T cells as described previously. The lentiviruses thus produced were harvested and used to T24 cells for 48 h. Finally, the infected cells were grown in 200 μl of 2 μg/ml puromycin. Ten days later, the surviving cells were amplified, and their identities were verified by Western blot. The sgRNA sequences (designed via at http://crispor.tefor.net/) were, forward, 5′-GGATCCTGAACTCCTTAGCA-3′, and reverse, 5′-TGCTAAGGAGTTCAGGATCC-3′.

### Cell viability assay

Cell proliferation ability was measured using Cell Counting Kit-8 (Beyotime, China). Each group of cells (2000 cells/well) was plated on 96-well plates. After 24 h, 48 h, or 72 h, each well was treated with 10 µL of CCK-8 reagent and then incubated at 37 °C for 2 h post-treatment, and then, the plates were read at 450 nm using a microplate reader.

### Colony formation and soft agar assays

To measure the long-term proliferation rates of cells, cells (1000 cells/well) were grown on 6-well plates for 14 days. Then, the cells were stained with 0.2% crystal violet for 30 min. After washing the cells with water, images were captured, and the number of clones was counted. The number of cells that formed colonies in soft agar was also counted to evaluate the anchorage-independent growth capacity of the cells. Briefly, medium containing 1.4% agarose was coated onto 6-well plates to form the bottom layer. Cells (5 × 10^4^ cells/well) were mixed with culture medium containing 0.7% agarose and added atop the bottom later. After 21 days of incubation, at which time the colonies were detected with the naked eye, the formatted colonies were counted, and the data were plotted.

### Transwell migration assays

To determine the migration ability of cells under different conditions, 500 µL of growth medium was added to 24-well plates, and 20% FBS was added as the chemotactic factor. Cells (1 × 10^4^) were suspended in 100 μL of serum-free medium and added to the Transwell insert in 24-well plates. After 24 h of maintenance in culture, T24 cells that did not migrate through the pores of the insert were removed with a cotton swab. The cells that migrated to the lower side of the insert filter were fixed with 5% glutaraldehyde for 10 min and then counterstained with 1% crystal violet in 2% ethanol for 20 min. The upper layer of cells was carefully wiped off, fixed, stained and photographed, and the number of tumor cells that entered the lower chamber was counted.

### Western blotting

RIPA buffer (1% NP-40, 0.5% sodium deoxycholate, 0.1% SDS, 10 μg/mL aprotinin, 10 μg/mL leupeptin, and 1 mM phenylmethylsulfonyl fluoride) was applied to prepare cell lysates. A Bradford protein assay kit (Beyotime, China) was used to determine the protein concentration. Subsequently, 20 µg of protein was subjected to 12% SDS‒PAGE and then electrotransferred onto polyvinylidene difluoride (PVDF) membranes (Millipore, Cat# IPVH00010, Merck KgaA, Darmstadt, Germany). The membranes were blocked with 5% nonfat milk for 1 h at room temperature before primary antibodies were added to the membranes, which were then incubated overnight at 4 °C. Then secondary antibodies were added to the membranes, which were incubated for 1 h at room temperature. The immunoreactive protein signal was developed with an Immobilon Western Chemiluminescent HRP Substrate Kit (Merck KgaA, Minneapolis, MN, USA).

### Coimmunoprecipitation (Co-IP) assay

A Pierce Co-Immunoprecipitation Kit (Thermo Fisher, USA) was used to verify the physical interaction between UCHL3 and CTNNB1. Briefly, transfected 293 T cells were lysed in ice-cold IP lysis buffer (30 mM Tris–HCl (pH 7.4), 150 mM NaCl, 1% NP-40, 10 µg/mL aprotinin, 10 μg/mL leupeptin, and 1 mM phenylmethylsulfonyl fluoride) for 5 min. After centrifugation, the cell debris was discarded, and the cell supernatant was retained. G-agarose beads (Smart-Lifesciences, Changzhou, China) precoated with the indicated antibodies were added to the supernatant with lysed cells, which was incubated at 4 °C. Six hours later, the co-IP proteins were subjected to Western blot analysis.

### Deubiquitination analysis

To measure the effect of UCHL3 on CTNNB1 deubiquitination, in vitro a deubiquitination analysis was performed. HEK293 T cells were transfected with plasmids, and at 24 h post-transfection, the cell culture medium was replaced with fresh culture medium containing 10 μmol MG132. At 36 h posttransfection, cells were washed with PBS and lysed with an equal volume of SDS lysis buffer (10% SDS in PBS). The lysates were heated to 95 °C, and then, a twofold volume of modified RIPA buffer (50 mM Tris–HCl (pH 7.4), 150 mM NaCl, 1 mM EDTA and protease inhibitor) was added to the lysates. Then, the lysates were cooled on ice for 30 min and centrifuged at 12,000×*g* for 15 min at 4 °C. Finally, the supernatant was subjected to anti-HA immunoprecipitation and Western blot analysis.

### Luciferase reporter assay

Five nanograms of TCF/LEF1-Luc vectors (Yeasen, China) were introduced into T24 cells or 293 T cells (1 × 10^5^) with the indicated dose of constructed UCHL3-Flag vectors. Forty-eight hours later, using a dual-luciferase assay kit (Promega), the luciferase activity was recorded using a Promega luciferase system.

### Immunofluorescence analysis

HEK293 T cells were cultured on coverslips and transfected with the HA-CTNNB1 and Flag-UCHL3 expression plasmids. After 36 h, the cells were fixed with 4% paraformaldehyde and permeabilized with 0.1% Triton X-100 in PBS. Then, the cells were washed with PBS and blocked with 5% BSA in PBS for 45 min. To visualize the expression of Flag-UCHL3 and HA-CTNNB1, mouse anti-Flag antibody and rabbit anti-HA antibody were used as primary antibodies, and mouse Alexa594- and rabbit Alexa488-conjugated antibodies (Invitrogen) were used as secondary antibodies. DAPI was used to stain the nuclei. Finally, the coverslips were observed and digitally photographed using a confocal microscope with a 60 × oil objective.

### Cycloheximide-chase (CHX) assay

Cells were treated with CHX (40 μM) at the indicated time points 48 h after transfection. Cell lysates were analyzed using standard western blotting.

### Animal study

*Upk2-Cre* and *Uchl3*^*fl/fl*^ mice were purchased from the Model Animal Research Center of Nanjing University. *Upk2-Cre*; *Uchl3*^*fl/fl*^ and *Uchl3*^*fl/fl*^ male mice were generated by breeding*.* After 2 weeks of acclimation, the mice (5 weeks old) were administered tamoxifen (75 mg/kg) for 5 days. Three weeks later, the mice were continuously fed drinking water with or without 0.05% BBN. The mouse weights were monitored every 3 days. Mice were sacrificed at the 26^th^ week, and tumor weights and volumes were recorded. All mice were also subjected to pathological examination. Survival percentage was also evaluated in tamoxifen-treated mice. Immunohistochemistry of tumor sections from mice was conducted as mentioned below. BALB/c mice aged 5–6 weeks were purchased from the Experimental Animal Center of Wuhan University (Wuhan, China). All mice were grown under SPF conditions with a 12:12 h dark:light cycle. Sufficient food and water were provided throughout the day. Animal ethics approval was granted by the University of Wuhan University. After 21 days, the mice were euthanized by CO_2_ asphyxiation, and the implanted tumors were resected, and their weight and volume were measured. The tumor tissues were fixed with 4% paraformaldehyde (PFA). Five-micron-thick sections were prepared for immunohistochemistry analysis and hematoxylin and eosin (HE) staining.

### Immunohistochemical analysis

Immunohistochemistry was performed as previously described. Briefly, slides were treated with 10% H_2_O_2_ and permeabilized with 0.1% TX-100. Next, primary antibodies against Ki67, CTNNB1, and LEF1 were added to slides to enable their binding to target proteins at 37 °C overnight, and then, the proteins were immunoblotted with secondary antibodies. Next, the proteins were visualized with an Avidin–Biotin Complex (ABC) staining systems (Vector Laboratories, Inc. USA) following the manufacturer’s instruction. An immunohistochemical analysis was also performed with a tissue microarray carrying tumoral (n = 176) and noncancerous tissues (n = 8) from individuals suffering from bladder cancer (Xian Alenabio).

### HE staining

The prepared tissue sections were deparaffinized by consecutive treatment with xylene and different concentrations of alcohol for 5 min for each immersion. Mayer hematoxylin (Wako, Japan) was used for nuclear counterstaining, and counterstaining was conducted by immersing each section in an eosin solution (1% eosin). The sections were dehydrated and hyalinized in alcohol before visualization with a microscope (Olympus Tokyo, Japan).

### Database analysis

The GEPIA and PROGgeneV2 database were used to analyze UCHL3 expression and determine its prognostic significance. The GSE31684 dataset was used to verify the prognostic relevance of UCHL3 expression. The clinical relevance of UCHL3 in patients with bladder cancer was analyzed by Sanchez-Carbayo Bladder 2 and Dyrskjot Bladder 3. We obtained the Sanchez and Dyrskjot datasets from Oncomine (https://www.oncomine.org), the data originated from NMIBC/MIBC patients [23, 24]. We utilized log-rank test and Cox regression analyses for Kaplan–Meier curves.

### Statistical analysis

Statistical significance was evaluated using GraphPad Prism software (GraphPad, USA). The data are expressed as the mean ± SEM. Student’s t test was performed to analyze the difference in data between two groups. One-way analysis of variance (ANOVA) with Bonferroni post hoc analysis was used to verify a difference in data obtained from multiple groups. P < 0.05 was considered statistically significant.

## Results

### Overexpressing UCHL3 boosts the proliferative, migratory and invasive potential of bladder cancer cells

**Fig. 1:**
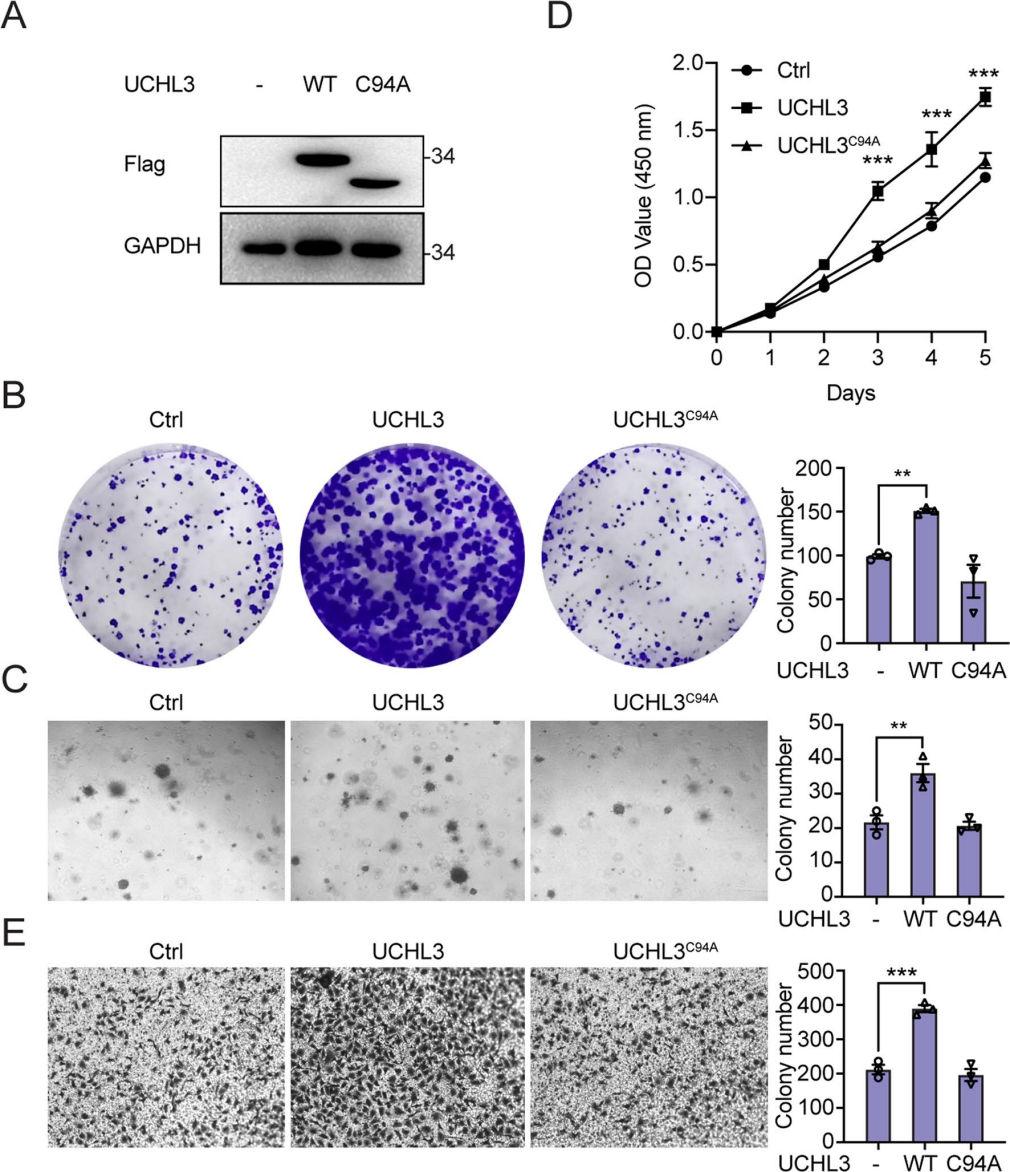
Overexpressing UCHL3 boosts the proliferative and migratory potential of bladder cancer cells. **A** The expression of Flag-UCHL3 and Flag-UCHL3^C94A^ was assessed by Western blotting. **B** Colony formation assays demonstrated the proliferation ability of Flag-UCHL3-overexpressing, Flag-UCHL3^C94A^-overexpressing and control EJ cells. The number of clones was counted and plotted. **C** Soft agar assays were performed to reveal the anchorage-independent growth ability of the Flag-UCHL3-overexpressing, Flag-UCHL3^C94A^-overexpressing and control EJ cells. The colony numbers are expressed as the means ± standard errors of triplicate assays. **D** A CCK-8 assay was conducted to examine the growth and proliferation ability of Flag-UCHL3-overexpressing, Flag-UCHL3^C94A^-overexpressing and control EJ cells. **E** Transwell migration assay. The migration ability of Flag-UCHL3-overexpressing, Flag-UCHL3^C94A^-overexpressing and control EJ cells was measured. *P < 0.05; **P < 0.001; ***P < 0.001 compared with controls

To gain insight into the biofunction of UCHL3 during bladder cancer development and progression, we forced the stable expression of UCHL3 in EJ cells. In addition, considering the deubiquitinating activity of UCHL3, a Ub hydrolase-deficient mutant (UCHL3^C94A^) was also transfected into EJ cells to establish a stable deubiquitinase-null UCHL3^C94A^ mutant cell line [22]. The overexpression of UCHL3 and UCHL3^C94A^ in EJ cells was confirmed by Western blotting (Fig. 1A). Next, a series of functional assessments were performed. As shown in Fig. 1B, D, colony formation assays and cell growth assays showed that ectopically expressed UCHL3 facilitated the EJ cell colony formation and pled to higher viability than that of wild-type EJ cells; however, UCHL3^C94A^ transfection into EJ cells resulted in no marked change in EJ cell colony formation ability or proliferation rate. Moreover, a soft agar colony formation assay showed a similar result: stable UCHL3 overexpression, but not UCHL3^C94A^ expression, notably increased the EJ cell invasion rate (Fig. 1C). In addition, UCHL3 overexpression promoted EJ cell migration, but no alteration in EJ cell migration was observed when the deubiquitination-catalyzing site in UCHL3 was mutated (Fig. 1E). Therefore, UCHL3 increased EJ cell proliferation, viability, migration and invasion, and these effects depended on its deubiquitinating activity.

### Abrogation of UCHL3 impairs the proliferation and migration abilities of bladder cancer cells

**Fig. 2:**
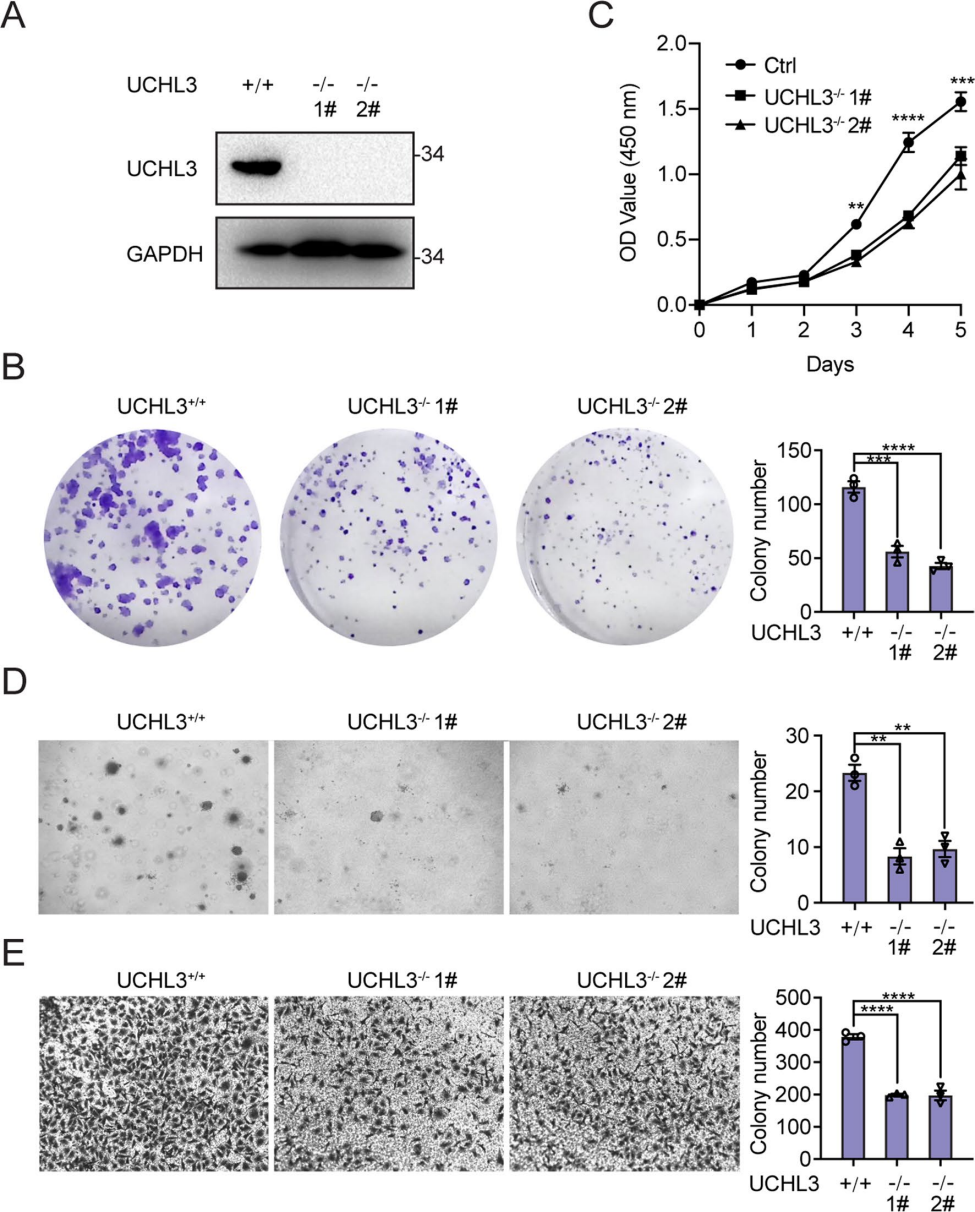
Depletion of UCHL3 causes proliferative and migratory defects in bladder cancer cells. **A** UCHL3-deficient cells were assessed by Western blotting. **B** Colony formation assays. **C** A CCK-8 assay was conducted to examine the growth and proliferation ability of UCHL3-deficient cells and control cell lines. **D** Soft agar assays were performed to reveal the anchorage-independent growth ability of UCHL3-deficient cells and control cell lines. **E** Migration ability of UCHL3-deficient cells and control cell lines was measured via Transwell migration assay. *P < 0.05; **P < 0.001; ***P < 0.001 compared with controls

To further determine the physiological function of UCHL3, UCHL3 was knocked out of T24 bladder cancer cells via the CRISPR‒Cas9 gene-editing technique. The impact of UCHL3 deficiency on cell growth and migration was assessed by colony formation, soft agar, CCK-8 and Transwell assays. A UCHL3-deficient T24 cell line was constructed, and its establishment based on the CRISPR–Cas9 strategy was confirmed by Western blotting (Fig. 2A). As illustrated in Fig. 2B, fewer colonies of T24 cells were observed after UCHL3 abrogation, which resulted in a reduced T24 cell proliferation rate. Moreover, we performed a CCK-8 assay in which UCHL3-deficient T24 cells also proliferated at a lower rate than the parental T24 cells (Fig. 2C). Subsequently, we performed soft agar colony formation assays to verify that UCHL3 deficiency markedly decreased the anchorage-independent growth of T24 cells (Fig. 2D). Furthermore, UCHL3-deficient T24 cells exhibited lower migratory activity (Fig. 2E). Thus, UCHL3 deficiency impairs T24 cell proliferation and migratory function in vitro.

### UCHL3 activates the Wnt signaling pathway

**Fig. 3:**
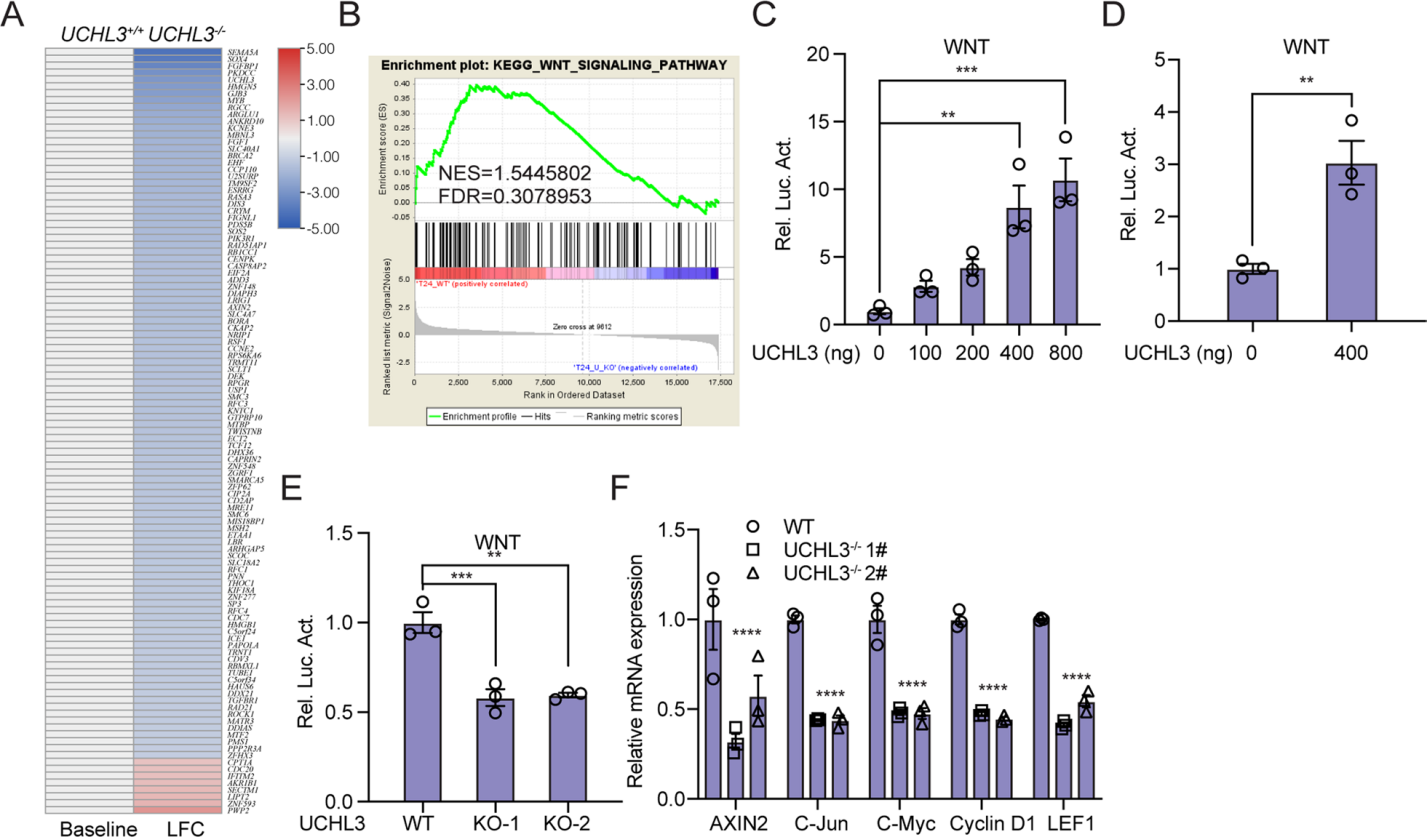
UCHL3 activates the Wnt signaling pathway. **A** Heatmap showing the significantly differentially expressed genes in UCHL3-deficient T24 cells and wild-type T24 cells. **B** Kyoto Encyclopedia of Genes and Genomes (KEGG) pathway enrichment analysis was conducted to assess the different signaling pathways between wild-type cells and UCHL3-knockout cells. **C**, **D** UCHL3 increases the transcriptional activity of the Wnt signaling pathway. Wnt reporter firefly luciferase plasmid (200 ng), CMV (10 ng) and the indicated amounts of UCHL3 plasmid were transfected into 293 T cells. Reporter assays were performed 48 h after transfection, and the results are presented as Wnt/CMV luciferase activity. **E** Luciferase assays were performed to reveal the influence of UCHL3 loss on the Wnt pathway in T24 cells. **F** UCHL3 loss inhibited the transcription of Wnt-targeted genes in T24 cells. The expression levels of the indicated Wnt-targeted genes were examined by RT‒PCR in UCHL3^−/−^ T24 cells and wild-type T24 cells. **P < 0.01; ***P < 0.001; ****P < 0.0001 compared with controls

To identify the potential mechanism underlying UCHL3-driven malignant behaviors in bladder cancer, we performed RNA-seq analysis using the UCHL3-deficient and WT T24 cell lines. We analyzed the differentially expressed genes in UCHL3-deficient T24 cells and wild-type T24 cells using a heatmap and performing a gene set enrichment analysis (GSEA). As visualized by the heatmap shown in Fig. 3A, we found a marked difference in the expression of genes between the two types of cells. The GSEA of these differentially expressed genes revealed that UCHL3-related differentially expressed genes were enriched in the Wnt signaling pathway (Fig. 3B). Considering the bioinformatics results in combination, we hypothesized that UCHL3 regulates the WNT signaling pathway. Subsequently, to test this hypothesis, we evaluated whether UCHL3 influences TCF/LEF1-mediated transcription by performing luciferase reporter assays. As depicted in Fig. 3C, UCHL3 triggered the gradual enhancement of Wnt-mediated luciferase activity in a dose-dependent manner. A significant enhancement was measured when T24 cells were transfected with TCF/LEF1-Luc vectors and 400 ng UCHL3-overexpressing vectors (Fig. 3D). Moreover, we examined the downregulation of TCF/LEF1-Luc activity in T24 cells when UCHL3 was depleted (Fig. 3E). Most importantly, the transcription of *AXIN2*, *C-Jun*, *C-Myc*, *Cyclin D1*, and *LEF1* was also downregulated in UCHL3-deficient T24 cells (Fig. 3F). Considering these findings, we believe that UCHL3 is important for regulating the activity of the WNT pathway. All the data suggested that UCHL3 triggered the activation of the Wnt signaling pathway.

### UCHL3 interacts with and stabilizes CTNNB1

**Fig. 4:**
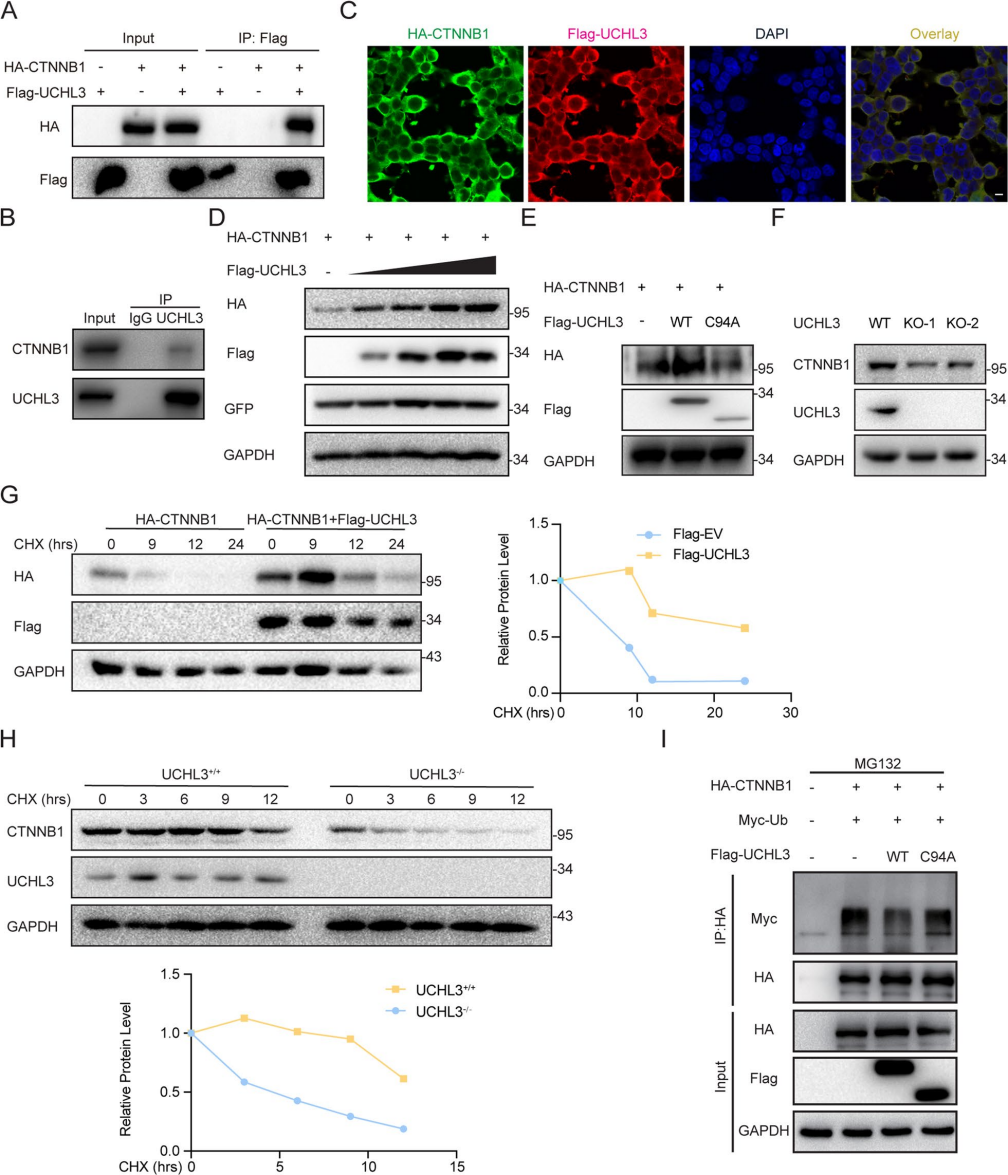
UCHL3 interacts with and stabilizes CTNNB1. **A** Immunoprecipitation with anti-Flag. HA-CTNNB1 vectors were coexpressed into 293 T cells with Flag-UCHL3 vectors. **B** Immunoprecipitation with anti-CTNNB1 followed by immunoblotting with anti-UCHL3 in T24 cells. **C** Colocalization of HA-CTNNB1 and Flag-UCHL3 in 293 T cells. Bar = 10 µm. **D** Western blot analysis of the effect of increasing amounts of UCHL3 (0, 200, 400, 600 and 800 ng) on CTNNB1 expression in 293 T cells. **E** Western blotting with the HA antibody was performed to measure CTNNB1 expression in T24 cells when HA-CTNNB1-overexpressing vectors and Flag-UCHL3-overexpressing vectors or Flag-UCHL3^C94A^-overexpressing vectors were cointroduced. **F** Western blot analysis of CTNNB1 expression in UCHL3-deficient T24 cells. **G**, **H** Western blot analysis of CTNNB1 expression stability in the presence or absence of UCHL3 (293 T or T24) after treatment with cycloheximide (CHX) (50 μg/ml) as measured at the indicated time points. **I** Western blot analysis of CTNNB1 ubiquitination in the presence of Flag-UCHL3 or the Flag-UCHL3^C94A^ mutant

Hyperactivated Wnt signaling contributes to an elevated cytosolic β-catenin protein level. Hence, we evaluated the interaction between UCHL3 and CTNNB1. Immunoblotting of cell lysates showed that UCHL3 coimmunoprecipitated with CTNNB1 when Flag-UCHL3 and HA-CTNNB1 were coexpressed in 293 T cells (Fig. 4A). To verify the interaction between UCHL3 and CTNNB1 in bladder cancer cells, the T24 cell line was selected for an endogenous immunoprecipitation (IP) analysis. The results showed that UCHL3 binds endogenous CTNNB1 under pathological conditions (Fig. 4B). Similar findings were observed via immunofluorescence analysis, which showed the colocalization of UCHL3 and CTNNB1 in the cytosol of 293 T cells (Fig. 4C). These results suggest that UCHL3 might physically interact with CTNNB1 to trigger activation of the Wnt signaling pathway.

**Fig. 5:**
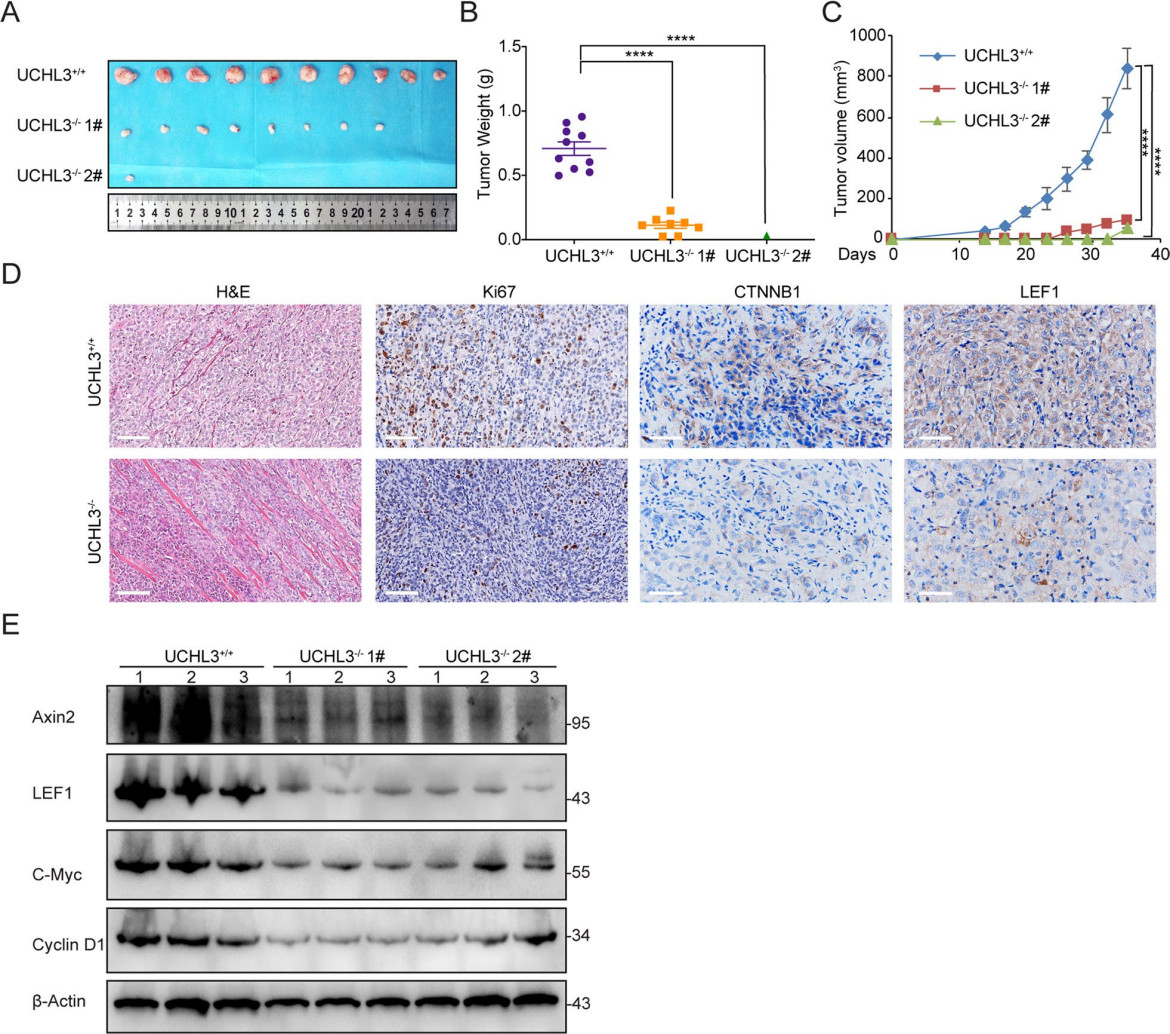
UCHL3 boosts bladder cancer tumorigenesis in vivo. **A** Images of tumor xenografts in nude mice on the 40th day after transplantation with UCHL3-deficient T24 cells and wild-type T24 cells. **B** Tumor weight in nude mice. **C** Growth curve of tumor xenografts. **D** Representative images (× 200) of H&E and IHC staining of the tumors. Bar = 100 µm. **E** Detection of related protein indices in tumor tissue samples via western blotting. ****P < 0.0001 compared with controls

Since UCHL3 is a DUB [18], its deubiquitinating activity is crucial to its function. We investigated whether UCHL3 impacts the stability of CTNNB1. We first cotransfected different amounts of Flag-UCHL3 (0, 200, 400, 600 and 800 ng) into 293 T cells with HA-CTNNB1. Western blot analyses revealed that HA-CTNNB1 expression gradually increased with the increased expression of FLAG-UCHL3, and this increase was dose dependent (Fig. 4D). To evaluate the effect of UCHL3 DUB activity on CTNNB1 stability, we overexpressed FLAG-UCHL3 or the FLAG-UCHL3^C94A^ mutant and found that the stability of FLAG-UCHL3 binding to HA-CTNNB1 depended on its ubiquitinase activity (Fig. 4E). Furthermore, to assess whether UCHL3 deficiency affects CTNNB1 protein levels, we measured CTNNB1 expression in the UCHL3-deficient cell line. As a result of UCHL3 deficiency, CTNNB1 expression was reduced in T24 UCHL3^−/−^ cells (Fig. 4F). We further evaluated the interaction of UCHL3 with CTNNB1 using a cycloheximide-chase (CHX) assay to verify that CTNNB1 protein levels are regulated by UCHL3 over time. As shown in Fig. 4G, the protein half-life of HA-CTNNB1 was obviously extended in the presence of FLAG-UCHL3 compared to that observed when an empty vector was used. Accordingly, UCHL3 deficiency decreased the CTNNB1 protein half-life (Fig. 5H). Consistent with these results, UCHL3 deficiency slightly accelerated CTNNB1 protein degradation in T24 cells (Fig. 4H). The most noted degradation mode of CTNNB1 was ubiquitin-dependent proteasomal degradation in the cytoplasm. Subsequently, we evaluated whether UCHL3 is able to catalyze the deubiquitination of CTNNB1. As shown in Fig. 4I, the level of ubiquitinated CTNNB1 was profoundly decreased by UCHL3 but not the UCHL3^C94A^ mutant. Taken together, UCHL3 promotes the stability of CTNNB1, and this effect depends on its deubiquitinating activity.

### *UCHL3 boosts bladder cancer tumorigenesis *in vivo

To validate the action of UCHL3 during bladder cancer in vivo, we established an in vivo nude mouse xenograft model via subcutaneous injection with UCHL3-deficient T24 cells and wild-type T24 cells. The size and weight of tumors were markedly decreased in the mice injected with UCHL3-deficient T24 cells compared with those injected with wild-type T24 cells (Fig. 5A, B). The volume of the tumors was measured throughout the experimental period. Clearly, UCHL3 deletion significantly delayed bladder carcinogenesis in vivo (Fig. 5C). HE staining and immunohistochemistry staining for Ki67, CTNNB1, and LEF1 was performed to measure their expression levels in tumor tissues. The outcome showed that UCHL3 ablation attenuated the pathological injury of tumor tissues and decreased the number of Ki67-positive cells (Fig. 5D, left). After UCHL3 ablation, the reduction in the number of CTNNB1-positive and LEF1-positive cells was also reduced (Fig. 5D, right). Western blot analysis showed that the protein levels of Wnt/β-Catenin downstream gene-encoded products were reduced in UCHL3-deficient tumor tissues, including LEF1, Axin2, C-myc and Cyclin D1, confirming that the abolition of UCHL3 inhibited WNT signaling pathway activation, which is consistent with our aforementioned experiments. Our data suggested that UCHL3 depletion reduced bladder tumorigenesis in a xenograft model and that this effect was at least partially dependent on CTNNB1 stabilization.

### Uchl3 deficiency leads to inhibited BBN-induced bladder tumorigenesis in mice

**Fig. 6:**
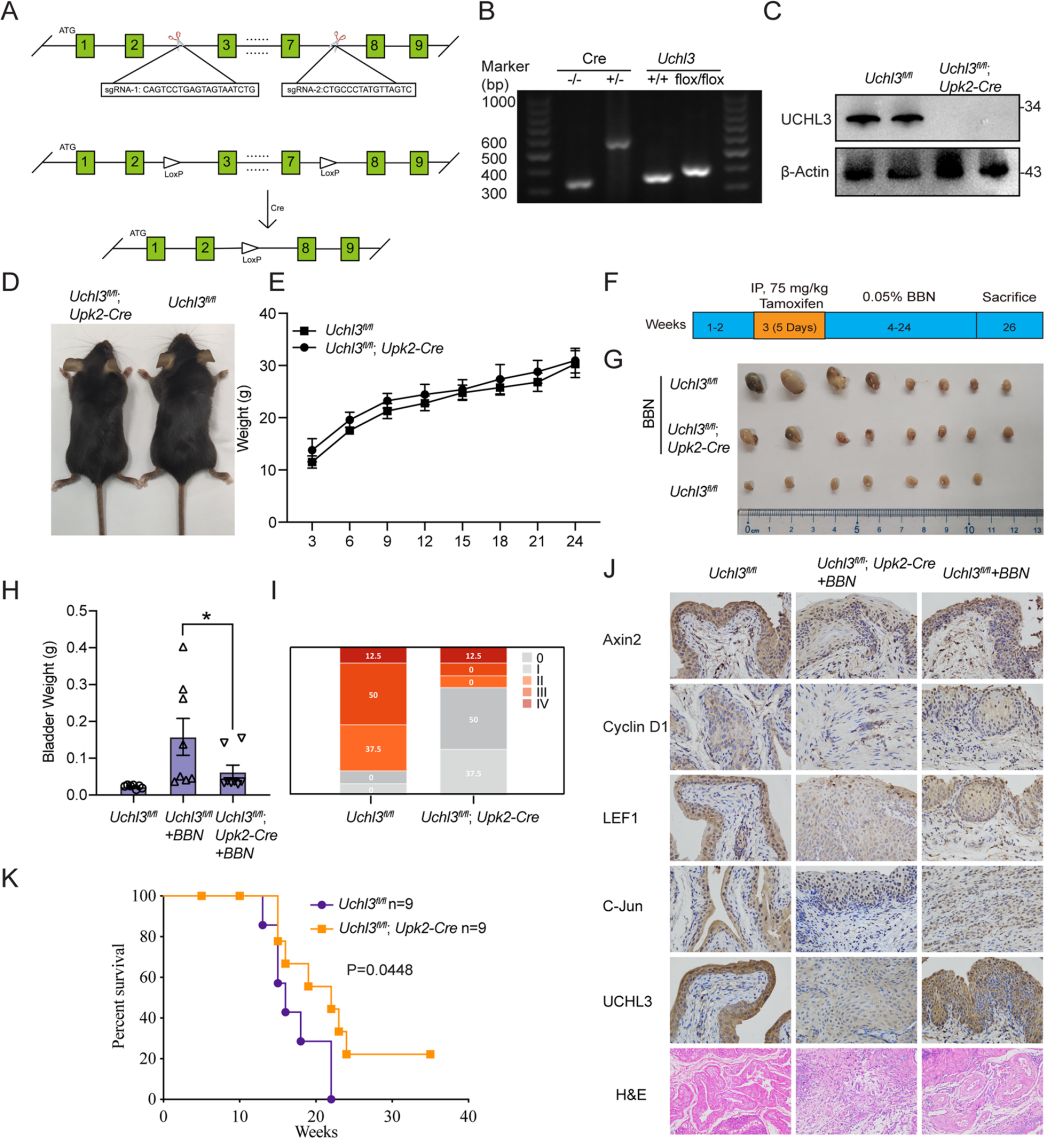
Uchl3 deficiency lessens BBN-induced bladder tumorigenesis in mice. **A** Schematic overview showing the strategy of *Uchl3* knockout using the CRISPR–Cas9 approach. **B** Representative image of electrophoresis of *Uchl3* in bladder tissues from *Uchl3*^*fl/fl*^ and *Uchl3*^*fl/fl*^;*Upk2-Cre* mice. **C** The protein levels of Uchl3 in bladder tissues from *Uchl3*^*fl/fl*^ and *Uchl3*^*fl/fl*^;*Upk2-Cre* mice were detected by Western blotting. **D** Representative image of *Uchl3*^*fl/fl*^ and *Uchl3*^*fl/fl*^;*Upk2-Cre* mice. **E** The weights of *Uchl3*^*fl/fl*^ and *Uchl3*^*fl/fl*^;*Upk2-Cre* mice were measured every 3 days. **F** Schematic overview showing BBN administration during the experiment to establish the bladder cancer model. **G** Representative images of BBN-induced bladders removed from *Uchl3*^*fl/fl*^ and *Uchl3*^*fl/fl*^;*Upk2-Cre* mice after sacrifice. **H** Bladder weight of *Uchl3*^*fl/fl*^ and *Uchl3*^*fl/fl*^;*Upk2-Cre* mice after the mice were sacrificed. **I** Statistical analysis of the pathological examination data of bladder tissues from *Uchl3*^*fl/fl*^ and *Uchl3*^*fl/fl*^;*Upk2-Cre* mice. **J** Immunohistochemical analysis of Axin2, Cyclin D1, Lef1, C-Jun and Uchl3 in bladder tissues from *Uchl3*^*fl/fl*^ and *Uchl3*^*fl/fl*^;*Upk2-Cre* mice. **K** Overall survival of *Uchl3*^*fl/fl*^* and Uchl3*^*fl/fl*^;*Upk2-Cre* mice in the BBN-induced bladder cancer model

To clarify the tumorigenic role of UCHL3 in bladder cancer in vivo, the CRISPR–Cas9 approach was employed to conditionally knock out *Uchl3* in the mouse bladder. The knockout strategy is shown in Fig. 6A. Vesical-specific *Uchl3*-deleted mice (*Uchl3*^*fl/fl*^;*Upk2-Cre*) were generated by crossing *UCHL3*^*fl/fl*^ conditional knockout mice with vesical-specific Upk2-Cre mice. Homozygous *UCHL3*^*fl/fl*^ and *Upk2-Cre* offspring were confirmed through genomic PCR (Fig. 6B). *Uchl3* deficiency in *Uchl3*^*fl/fl*^;*Upk2-Cre* mice was verified by Western blotting (Fig. 6C). A BBN-induced bladder cancer model was established to assess the effect of vesicular-specific *Uchl3* deletion on tumorigenesis. The procedure is shown in Fig. 6f. The target mice were fed with chow adapted to the conditions for one to two weeks following 75 mg/kg tamoxifen intraperitoneal injection for 5 days and water containing 0.05% BBN for 20 consecutive weeks. The mice were sacrificed on the 26th week, and the bladder tissue of the mice was removed. During 0.05% BBN treatment, the body weights of the mice were recorded once every three days. As shown in Fig. 6D, E, no significant weight reduction was observed in the *Uchl3*^*fl/fl*^;*Upk2-Cre* mice compared with *UCHL3*^*fl/fl*^ mice. Nevertheless, according to Fig. 6F, we successfully established a BBN-induced bladder cancer model and found that the deletion of UCHL3 reduced the rate of BBN-induced bladder cancerogenesis in the *Uchl3*^*fl/fl*^;*Upk2-Cre* mice compared with the control group (Fig. 6G). Specifically, the *UCHL3*^*fl/fl*^ group carried a larger weight than the *Uchl3*^*fl/fl*^;*Upk2-Cre* group (Fig. 6H). Furthermore, the bladders were graded pathologically. When compared with the *Uchl3*^*fl/fl*^ group, 87.5% of the tumors in the *Uchl3*^*fl/fl*^;*Upk2-Cre* group were in an early stage, while 87.5% of the tumors in the *Uchl3*^*fl/fl*^ group were Stage II or III (Fig. 6I). In addition, *Uchl3-*deficient mice presented with a higher survival rate than *Uchl3*^*fl/fl*^ mice with BBN-induced bladder cancer (Fig. 6K). These results suggested that Uchl3 knockout alleviated BBN-induced bladder tumorigenesis in vivo. Finally, the protein levels of Axin2, Ccnd1, Jun, Lef1, and Uchl3 in bladder tissues were measured using IHC staining, which showed marked decrease in WNT pathway signaling in *Uchl3-*deficient mice (Fig. 6J). In summary, Uchl3 tends to facilitate bladder cancer progression by stabilizing CTNNB1 and promoting WNT pathway activity in vivo.

### Amplification of UCHL3 is associated with worse prognosis

**Fig. 7:**
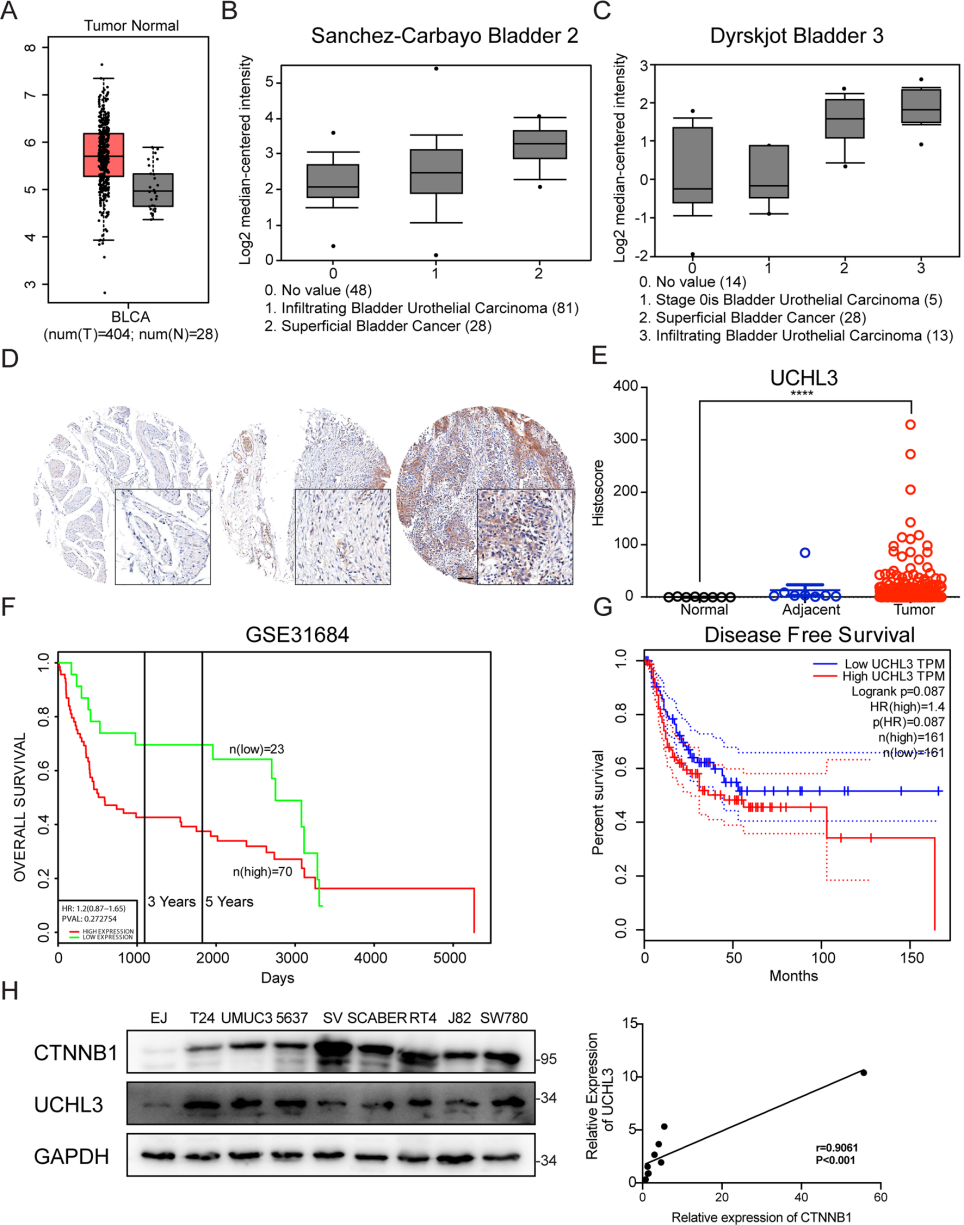
Amplification of UCHL3 was associated with worse prognosis. **A** The GEPIA database was employed to analyze UCHL3 expression in BLCA and normal tissues. **B** The Sanchez-Carbayo Bladder 2 database was employed to analyze UCHL3 expression in patients with no value, infiltrating bladder urothelial carcinoma, and superficial bladder cancer. **C** The Dyrskjot Bladder 3 database was employed to analyze UCHL3 expression in patients with no value, infiltrating bladder urothelial carcinoma and superficial bladder cancer. **D** Immunohistochemical analysis of UCHL3 was undertaken with a tissue microarray of human bladder cancer samples. Bar = 100 µm. **E** Quantitative histoscore of immunohistochemical staining for UCHL3. **F** The impact of UCHL3 on the overall survival of bladder cancer was evaluated via the GSE31684 dataset. **G** The GEPIA database was adopted to analyze the difference in disease-free survival among bladder cancer patients based on UCHL3 expression. **H** Western blots analyzing UCHL3 expression in several bladder cancer tissues

Having determined the biofunction of UCHL3 in bladder cancer, we assessed its clinical relevance. In line with its oncogenic role in bladder cancer in vitro and in vivo, UCHL3 was amplified in a TCGA cohort of bladder cancer patients via GEPIA software (Fig. 7A). Next, the bladder cancer dataset retrieved from Sanchez-Carbayo 2 and Dyrskjot Bladder 3 was analyzed, and UCHL3 expression was found to be progressively increased with tumor stage (Fig. 7B, C). Similarly, UCHL3 immunohistochemistry with a bladder cancer tissue microarray verified high UCHL3 expression relative to normal tissues (Fig. 7D, E). Notably, bladder cancer patients with high UCHL3 levels receive a grim prognosis (Fig. 7F, G). In several bladder cancer cell lines, high levels of UCHL3 protein were accompanied by high levels of CTNNB1 protein (Fig. 7H). All data validated an oncogenic action of UCHL3 during bladder carcinogenesis and tumor progression.

## Discussion

**Fig. 8:**
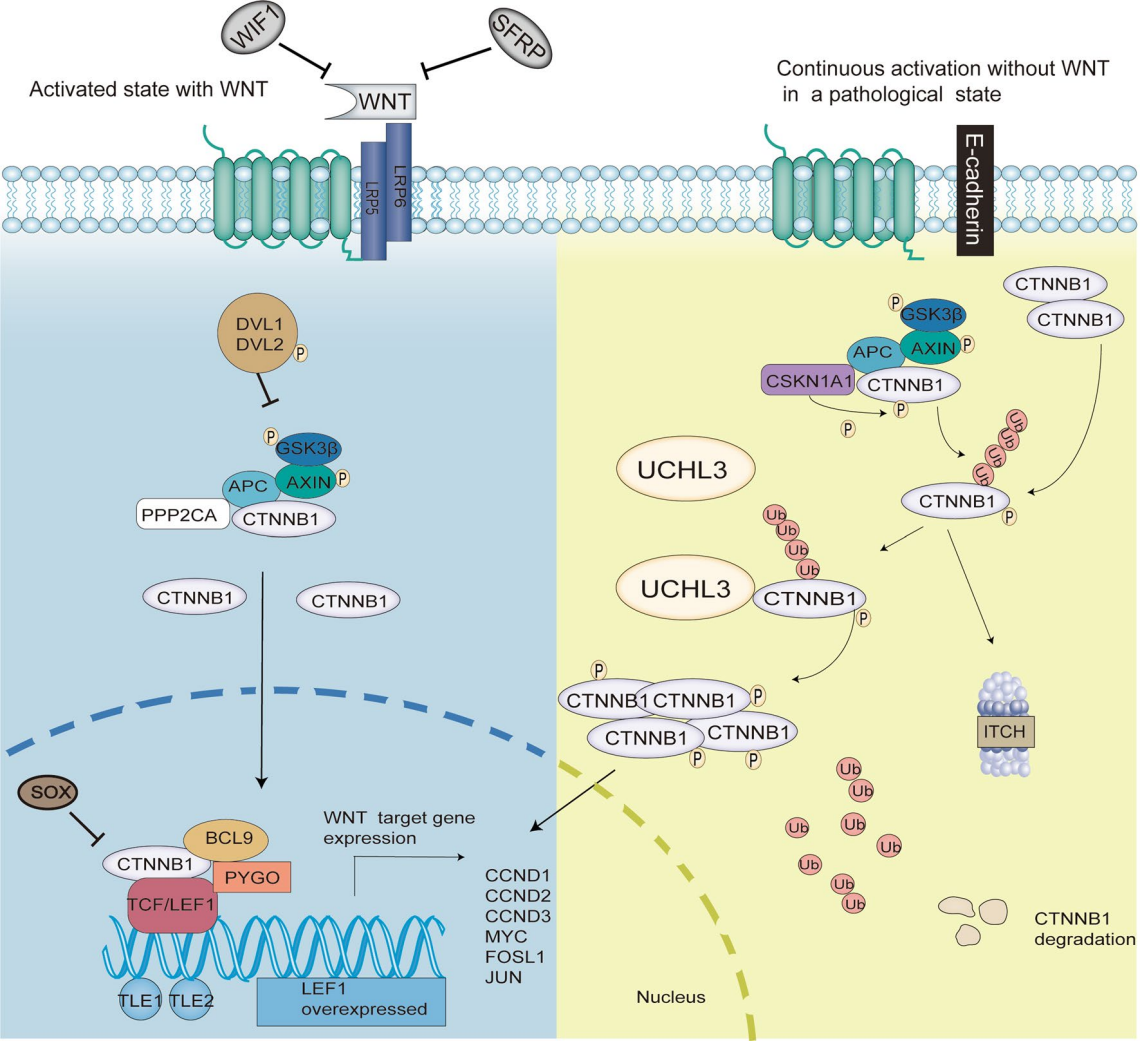
Schematic of the UCHL3/CTNNB1 signaling pathway

Bladder cancer is a common tumor and is also referred to as a highly malignant tumor. In addition to clinical diagnosis and therapeutic approaches, recent studies have focused on studying the molecular mechanisms underlying tumorigenesis and progression of bladder cancer. A myriad of studies have confirmed that DNA mutations or methylation can ectopically regulate the Wnt signaling pathway, resulting in deregulated protein expression, which may lead to bladder carcinogenesis and cancer progression [8, 25–28]. Various substances associated with the Wnt signaling pathway can be used as therapeutic targets for bladder cancer. The stability of CTNNB1 is one of the most important targets. In our work, we revealed that the expression of deubiquitinase UCHL3 was abundant in bladder cancer and associated with poorer clinical outcomes. Functionally, UCHL3 positively regulated bladder cancer cell proliferation and migration, and the depletion of UCHL3 resulted in tumor suppression in vitro and in vivo. Furthermore, UCHL3 stabilized CTNNB1 and subsequently triggered the oncogenic Wnt signaling pathway (Fig. 8). Our data demonstrated the tumor-promoting function of UCHL3 in bladder cancer, suggesting that targeting UCHL3 might be a potential approach for preventing bladder tumorigenesis and progression.

Previously, UCHL3 has been found to be robustly expressed in epithelial ovarian cancer and related to intra-abdominal metastases [29]. UCHL3 has been described as a tumor promoter that regulates DNA methylation, chromosome stability, and DNA repair in different types of malignancies [30–33]. UCHL3 encodes a protein characterized by deubiquitylating activity. Highly expressed UCHL3 is critical for aerobic glycolysis in pancreatic cancer because it stabilizes tumor promoter LDHA (lactate dehydrogenase A) expression [34]. UCHL3 maintains AhR protein stability and thereby confers cancer stem-like properties to non-small cell lung cancer cells, functioning as a tumor promoter [21]. In addition, our findings revealed that UCHL3 was abundantly expressed in bladder cancer, and this was associated with deleterious clinicopathological features of bladder cancer. As expected, overexpressing UCHL3 favored the proliferative and migratory activity of bladder cancer cells, and UCHL3 depletion caused reduced bladder cancer cell proliferation and migration. The effect of *Uchl3* knockout in vivo was also evaluated, and the results suggested it suppressed tumor growth. *Uchl3*-deficient mice were less prone to tumorigenesis and presented with a lower tumor burden. Thus, UCHL3 functions as a tumor promoter during bladder cancer occurrence and progression.

In terms of the underlying mechanism, bioinformatics analysis demonstrated that the Wnt signaling pathway is associated with UCHL3. Attenuation of Wnt-driven luciferase activity was observed in UCHL3-deficient T24 cells, and overexpressing UCHL3 enhanced Wnt-mediated luciferase activity. The downregulation of *AXIN2*, *C-Jun*, *C-Myc*, *Cyclin D1* and *LEF1* expression in UCHL3-deficient T24 cells validated a role for UCHL3 in Wnt signaling pathway activation. Wnt-driven events are important in human malignancies [7]. Wnt pathway hyperactivation is observed in many cancers [35]. After it is activated, CTNNB1 anchors to the actin cytoskeleton and may be critical for transmitting the contact-inhibiting signals that cause cells to stop dividing after an epithelial sheet is formed [36]. Mutations in this gene are causes of colorectal cancer, pilomatrixoma, medulloblastoma (MDB), and ovarian cancer [11, 37, 38]. Deubiquitylation of CTNNB1 has also been detected in cancers [39]. Interestingly, we found that UCHL3 interacted with CTNNB1, as shown by co-IP and colocalization assays with T24 cells. UCHL3 deficiency also reduced CTNNB1 expression in xenograft tumors and mouse bladders. Therefore, UCHL3 might suppress the ubiquitin-driven degradation of CTNNB1 and thereby trigger the Wnt signaling pathway, resulting in tumorigenic promotion.

Although the stabilization of UCHL3 depends on its deubiquitinating activity, UCHL3 directly deubiquitinates CTNNB1, and the sites of CTNNB1 that are deubiquitinated by UCHL3 need to be identified.

In summary, we showed that UCHL3 expression is amplified in bladder cancer and functions as a tumor promoter that enhances the proliferation and migration of bladder cancer cells in vitro and to bladder tumorigenesis and tumor progression in vivo. Furthermore, we revealed that UCHL3 stabilizes CTNNB1 expression, resulting in the activation of the oncogenic Wnt signaling pathway. Therefore, our findings strongly suggest that UCHL3 is a promising target against bladder cancer.

## Data Availability

The datasets generated/analyzed during the current study are available.
